# Supplementary material for: SYK Inhibition Potentiates the Effect of Chemotherapeutic Drugs on Neuroblastoma Cells In Vitro
Source: Cancers (Basel). 2019 Feb 10;11(2):202. doi: 10.3390/cancers11020202 (PMC6406899; doi:10.3390/cancers11020202)
Supplement: Supplementary file 1 [file cancers-11-00202-s001.pdf]

Supplementary Material

SYK Inhibition Potentiates the Effect of Chemotherapeutic Drugs on Neuroblastoma Cells *In Vitro*

Conny Tümmler, Gianina Dumitriu, Malin Wickström, Peter Coopman, Andrey Valkov, Per Kogner, John Inge Johnsen, Ugo Moens and Baldur Sveinbjörnsson

Table S1. Clinical features of neuroblastoma tumors.

		Number (%)
Age		
	<18 month	22 (52%)
	>18 month	20 (48%)
Gender		
	Female	22 (52%)
	Male	20 (48%)
INSS Stage		
	1	10 (24%)
	2	9 (21%)
	3	11 (26%)
	4	10 (24%)
	4s	2 (5%)
MYCN amplification		10 (24%)
1p deletion		9 (21%)
11q deletion		3 (7%)
17q gain		7 (17%)
Treated tissue		13 (31%)
Untreated tissue		26 (62%)
Information not available		3 (7%)

Table 2. Cell viability of SH-SY5Y and SK-N-BE(2) after treatment with 0.8 µM BAY 61-3606, chemotherapeutic drugs or combinations of both.

		**SH-SY5Y**			**SK-N-BE(2)**		
	Treatment	Cell viability (%) Mean ± SD	P value Drug vs. combination	P value BAY vs. combination	Cell viability (%) Mean ± SD	P value Drug vs. combination	P value BAY vs. combination
**48 h**	0.8µM BAY	51.26 ± 7.83			82.4 ± 8.79		
	Paclitaxel	87.71 ± 7.83			90.89 ± 7.86		
	Paclitaxel + BAY	31.63 ± 2.23	**<0.001**	**<0.001**	54.61 ± 4.12	**<0.001**	**<0.001**
	Cisplatin	82.33 ± 9.01			98.94 ± 6.48		
	Cisplatin + BAY	42.53 ± 2.96	**<0.001**	**0.001**	73.21 ± 5.16	**<0.001**	**<0.001**
	Doxorubicin	100.5 ± 9.59			102 ± 9.68		
	Doxorubicin + BAY	49.15 ± 6.39	**<0.001**	>0.999	76.06 ± 8.81	**<0.001**	0.167
	Temozolomide	107 ± 11.17			107.7 ± 3.65		
	Temozolomide + BAY	44.56 ± 3.83	**<0.001**	**0.018**	76.81 ± 3.85	**<0.001**	0.304
**72 h**	0.8µM BAY	45.38 ±7.87			82 ± 9.05		
	Paclitaxel	84.53 ± 4.6			88.29 ± 5.19		
	Paclitaxel + BAY	21.79 ± 1.28	**<0.001**	**<0.001**	38.07 ± 4.55	**<0.001**	**<0.001**
	Cisplatin	73.38 ± 5.89			73.99 ± 2.95		
	Cisplatin + BAY	36.68 ± 4.99	**<0.001**	**<0.001**	61.53 ± 4.99	**<0.001**	**<0.001**
	Doxorubicin	102.2 ± 8.37			101.3 ± 7.56		
	Doxorubicin + BAY	47.36 ± 6.6	**<0.001**	>0.999	75.18 ± 8.05	**<0.001**	**0.008**
	Temozolomide	107.7 ± 6.13			102.7 ± 4.28		
	Temozolomide + BAY	40.55 ± 2.79	**<0.001**	0.997	80.87 ± 6.6	**<0.001**	>0.999

Cell viability was measured by MTT assay after 48 and 72 h. The control was set as 100% viable cells. Data are presented as mean ± SD from at least three independent experiments. Using two-way ANOVA, a significant effect was observed for both treatment and between cell lines $p < 0.001$; Bonferroni's multiple comparison test was used to evaluate differences between treatments and p values < 0.05 were considered as statistically significant.

A

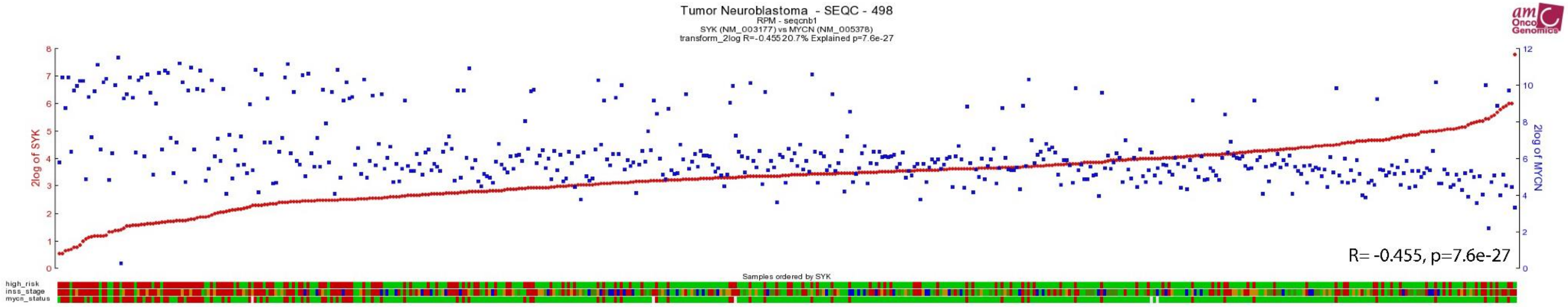

B

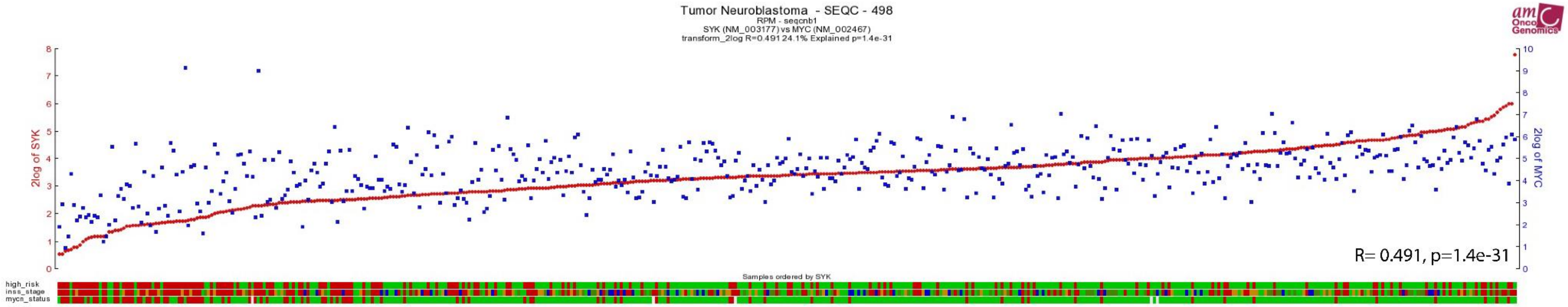

Figure S1. The expression of *SYK* is negatively correlated to *MYCN* but positively to *MYC* in neuroblastoma tissue. Gene expression data were analyzed using the R2 database http://r2.amc.nl. (**A**) Correlation of *SYK* and *MYCN* expression in neuroblastoma tissue using the SEQC dataset (n = 498). (**B**) Correlation of *SYK* and *MYC* expression in neuroblastoma tissue using the SEQC dataset (n = 498).

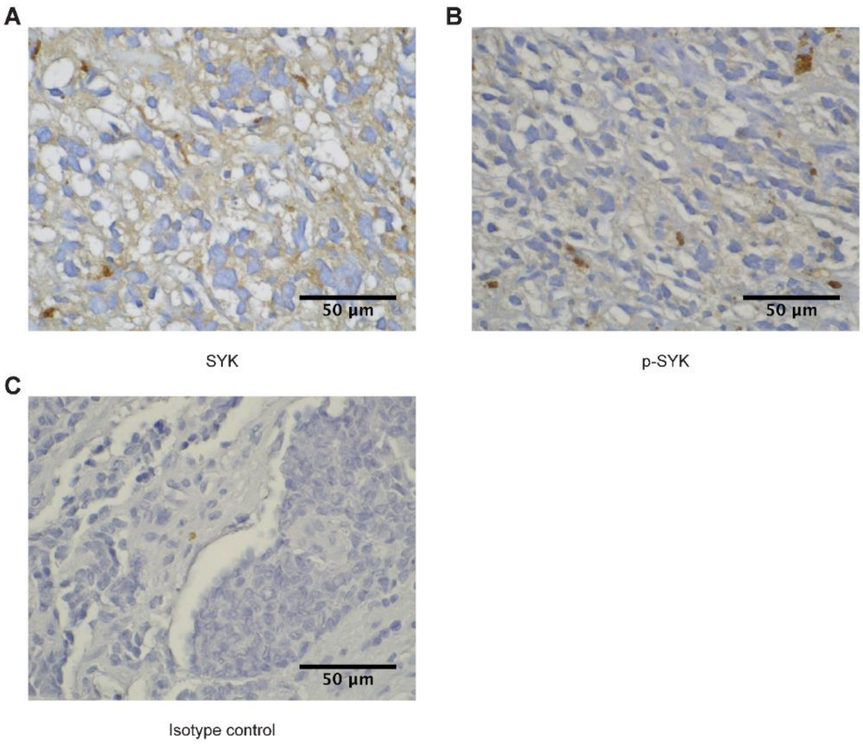

Figure S2. IHC of neuroblastoma tumors negative for SYK and p-SYK. Representative images of immunoperoxidase labeled tumor sections negative for SYK (**A**) and p-SYK (**B**). (**C**) Isotype control, where the primary antibody was replaced with the appropriate rabbit isotype antibody. Images were captured at a magnification of 900×.

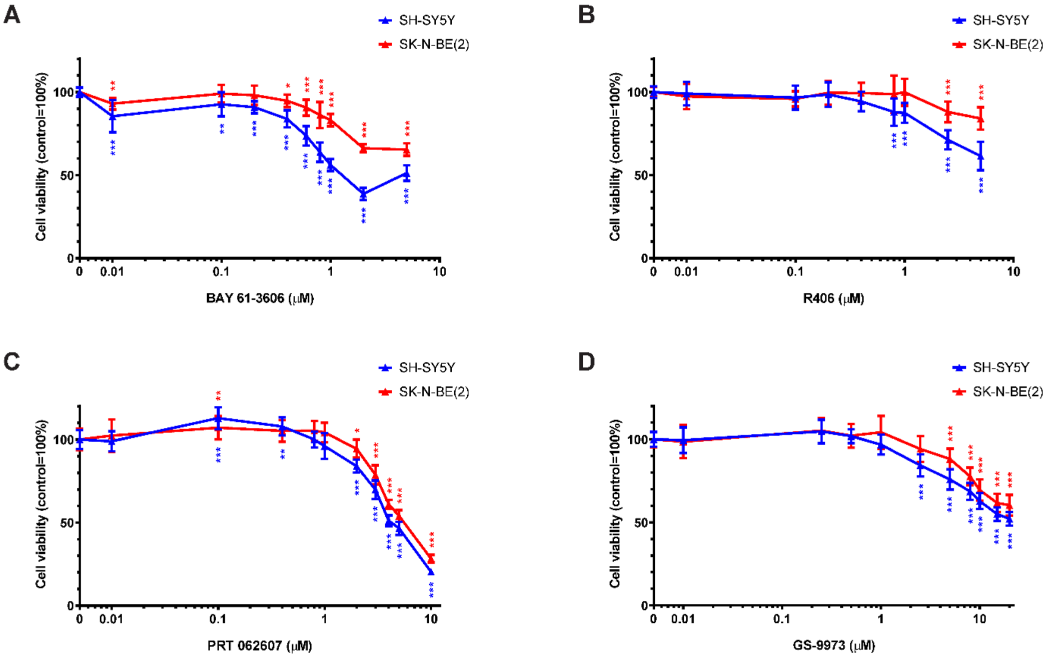

Figure S3. Inhibition of SYK decreases the cell viability of neuroblastoma cells. Cell viability was measured in SH-SY5Y and SK-N-BE(2) cells by MTT assay after 24 h incubation with increasing concentrations of the SYK inhibitors BAY 61-3606 (**A**) R406 (**B**) PRT 062607 (**C**) GS-9973 (**D**). The control was set as 100% viable cells. Data are presented as mean ± SD from three independent experiments. Using two-way ANOVA, a significant difference between cell lines and significant effect of the inhibitor $p < 0.001$ was seen. Dunnett's multiple comparison test was used to evaluate the difference between vehicle treated control cells and the various inhibitor concentrations* $p < 0.05$ ** $p < 0.01$ *** $p < 0.001$.

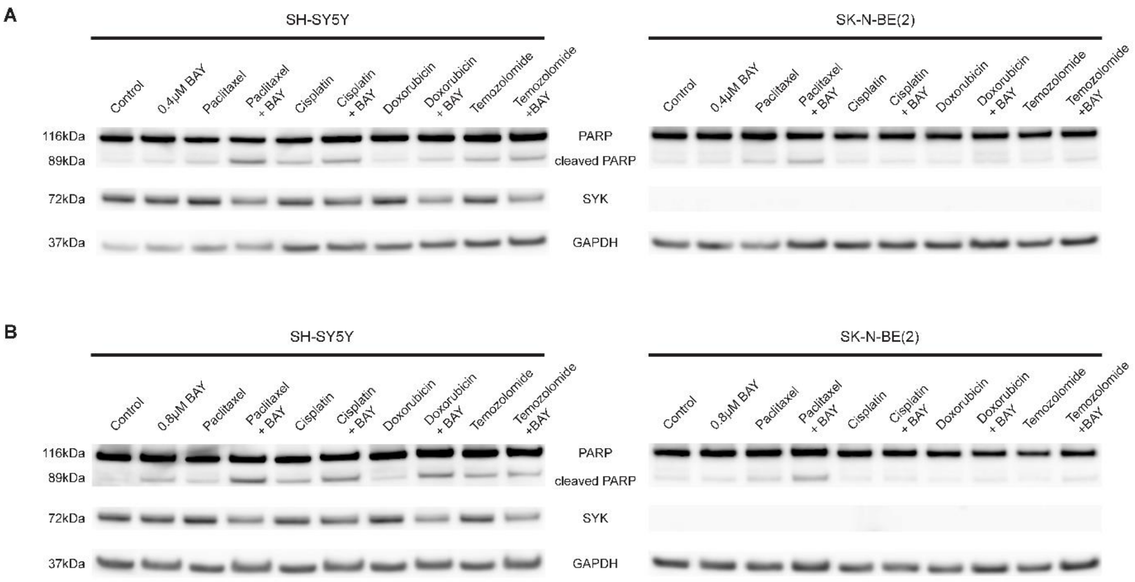

Figure S4. Combination of chemotherapeutic drugs and the selective SYK inhibitor BAY 61-3606 promotes PARP cleavage in neuroblastoma cells. PARP cleavage and SYK expression were determined by western blot after 48 h monotherapy or combinations of 0.4 µM (**A**) or 0.8 µM (**B**) BAY 61-3606, 20 nM paclitaxel, 5 nM doxorubicin, 100 µM temozolomide and cisplatin (1 µM or 3 µM for SH-SY5Y and SK-N BE(2), respectively).

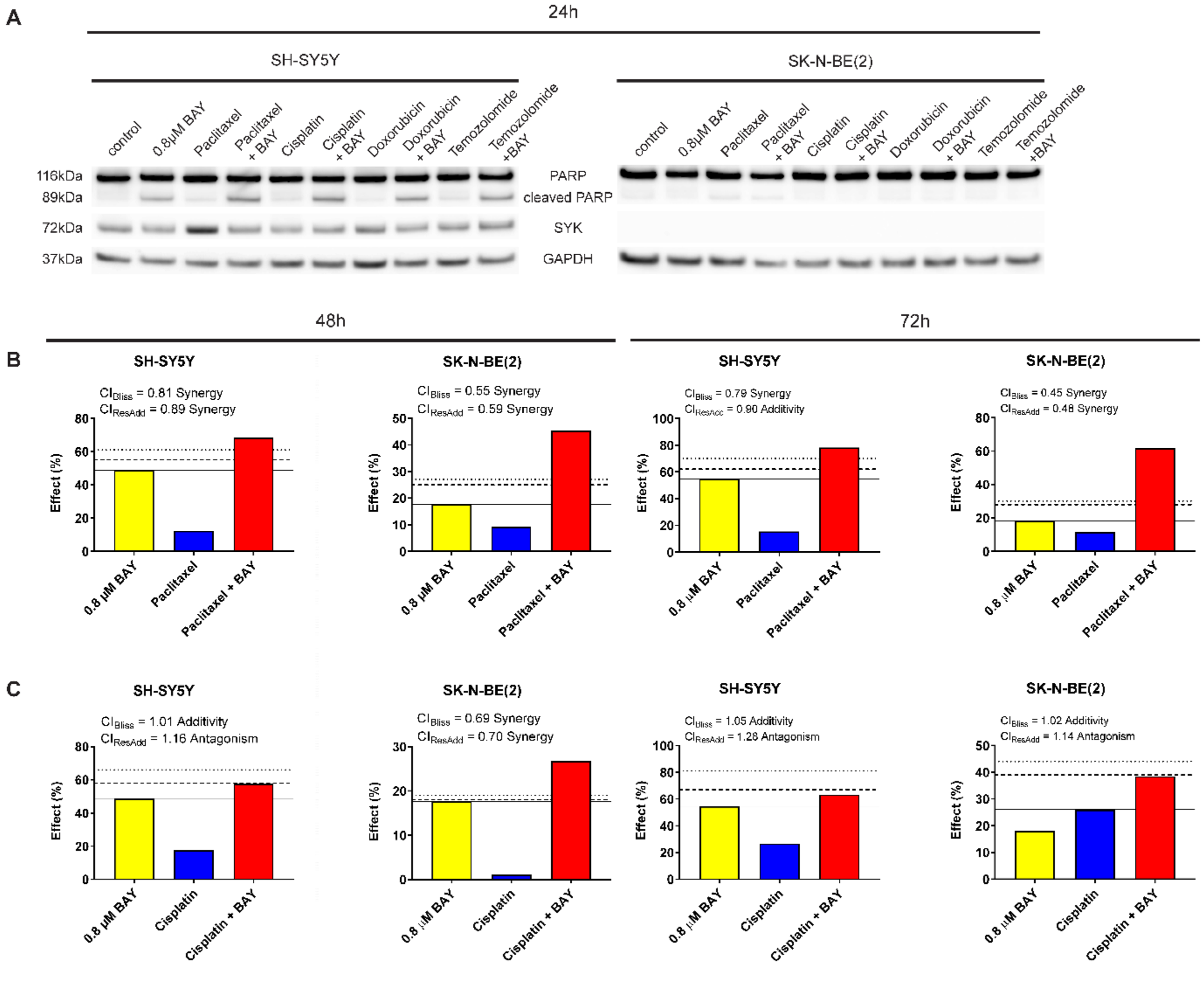

Figure 5. The selective SYK inhibitor BAY 61-3606 enhances the effect of chemotherapeutic drugs in neuroblastoma cells. (**A**) PARP cleavage and SYK expression were determined by western blot after 24 h monotherapy or combinations of 0.8 µM BAY 61-3606, 20 nM paclitaxel, 5 nM doxorubicin,

100 µM temozolomide and cisplatin (1 µM or 3 µM for SH-SY5Y and SK-N-BE(2), respectively). Illustration of drug combination effects for 0.8 µM BAY 61-3606 and paclitaxel (**B**) as well as 0.8 µM BAY 61-3606 and cisplatin (**C**) in SH-SY5Y and SK-N-BE(2) cells after 48 h and 72 h treatment. The continuous horizontal line indicates the effect of the highest single agent, the dashed line denotes expected additive effect calculated by the Bliss independence model, and the dotted line shows expected additive effect calculated by response additivity. Combination index (CI), given from the Bliss independence model and the response additivity, and effect are specified for each combination.

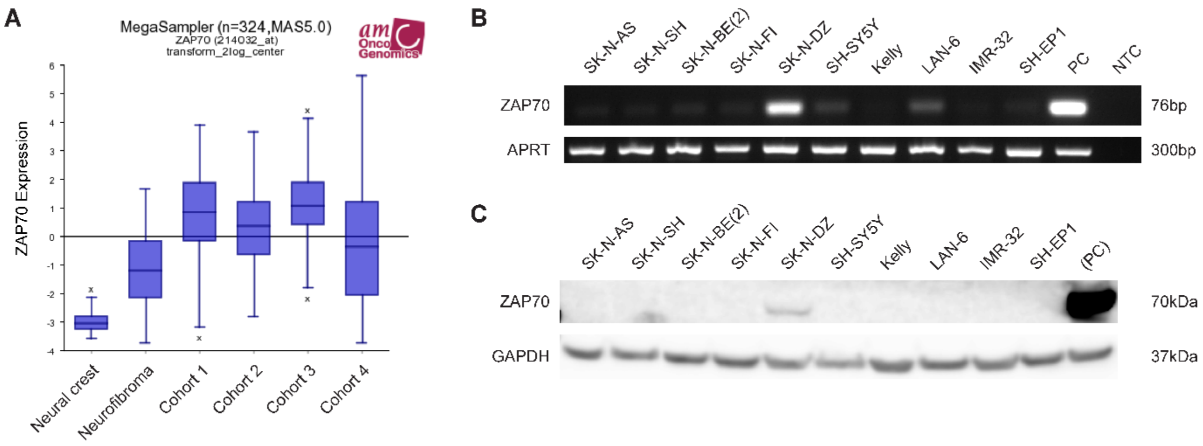

Figure S6. ZAP70 is expressed in at least one neuroblastoma cell line**.** Expression data were analyzed using the R2 database http://r2.amc.nl. (**A**) The expression of *ZAP70* was compared between neural crest (Etchevers n = 5), benign neurofibroma (Miller n = 86) and 4 neuroblastoma cohorts (cohort 1: Versteeg n = 88, cohort 2: Delattre n = 4, cohort 3: Hiyama n = 51, cohort 4: Lastowska n = 30). (**B**) RT-PCR analysis demonstrating the expression *ZAP70* mRNA in some of the examined neuroblastoma cell lines. Jurkat cells were used as a positive control (PC). NTC, no template control. (**C**) western blot of ZAP70 with Jurkat cells as a positive control.
